# Targeting polymorphonuclear myeloid‐derived suppressor cells in the immunosuppressive tumor microenvironment for cancer immunotherapy

**DOI:** 10.1002/mco2.602

**Published:** 2024-06-22

**Authors:** Shiqi Li, Xinghua Long

**Affiliations:** ^1^ Department of Laboratory Medicine Zhongnan Hospital of Wuhan University Wuhan China

## Abstract

Tumor‐driven immune suppression is a critical mechanism by which cancer cells evade the host immune system, leading to tumor growth and metastasis. The tumor immune microenvironment contains a large population of immune‐suppressing myeloid cells, which play a key role in tumor development and drug resistance to existing immunotherapy. Polymorphonuclear myeloid‐derived suppressor cells (PMN‐MDSCs) are important components of the immunosuppressive microenvironment. Uncovering the molecular mechanisms of PMN‐MDSCs and finding specific targets for PMN‐MDSCs to regulate tumor immune microenvironment is the focus and challenge of current immunotherapy. In a recent issue of Nature, Wang and colleagues revealed that CD300ld on PMN‐MDSCs is required for tumor‐driven immune suppression(1), this provided a new target for cancer immunotherapy, The study identified CD300ld as a novel, highly conserved tumor immunosuppressive receptor. CD300ld is highly expressed specifically on PMN‐MDSCs and is a key receptor in regulating the recruitment and immunosuppressant function of PMN‐MDSCs. Targeting CD300ld can reshape the tumor immune microenvironment by inhibiting the recruitment and function of PMN‐MDSCs, resulting in broad‐spectrum anti‐tumor effects. CD300ld target shows good safety, conservation, anti‐tumor effectiveness, and synergism with the Programmed death‐1 target, which is expected to become a new ideal target for tumor immunotherapy.

## Graphical abstract

The role of polymorphonuclear myelopoietic suppressor cells in immunosuppressive tumor microenvironment.

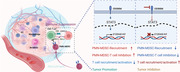

1

Tumor‐driven immune suppression is a pivotal mechanism through which cancer cells can escape detection and destruction by the host immune system, leading to unchecked tumor progression and metastasis. Within the tumor immune microenvironment, a substantial population of myeloid cells that exert immune‐suppressing effects significantly contributes to tumor evolution and resistance to current immunotherapies. Among these, polymorphonuclear myeloid‐derived suppressor cells (PMN‐MDSCs) represent a critical element of this immunosuppressive landscape. Elucidating the molecular underpinnings of PMN‐MDSCs and identifying specific therapeutic targets to modulate the tumor immune microenvironment remain central challenges and focuses within the realm of immunotherapy. In a recent edition of Nature, Wang et al. shed light on the indispensable role of CD300ld in facilitating tumor‐mediated immune suppression in PMN‐MDSCs,^1^ which provided a new target for cancer immunotherapy. This study highlights CD300ld as an innovative and highly conserved receptor implicated in the suppression of immune responses by tumors.^2^ Targeting CD300ld can reshape the tumor immune microenvironment by inhibiting the recruitment and inhibitory function of PMN‐MDSCs, resulting in broad‐spectrum antitumor effects. The targeting of CD300ld has yielded positive results in terms of safety, preservation of healthy tissue, antitumor efficacy, and compatibility with existing PD1‐based therapies, laying the groundwork for it to be considered an ideal candidate for novel immunotherapeutic approaches.

Immune checkpoint blockade (ICB) therapies, including PD‐1/PD‐L1/CTLA‐4 inhibitors, have made substantial advances in tumor treatment in recent years. Despite their success, the response to these ICBs can be strikingly variable among patients with different tumor types. Notably, a substantial subset of patients exhibit nonresponsiveness or even resistance to these treatments, underlining the need for more universally effective immunotherapeutic strategies. The investigation of CD300ld cells represents a promising development in this context, potentially offering broader efficacy across a range of tumor profiles. The tumor immune microenvironment contains a large population of immune‐suppressing myeloid cells that play key roles in tumor development and drug resistance to existing immunotherapies. Myeloid‐derived suppressor cells (MDSCs) are pivotal in fostering tumor growth by orchestrating immune responses that favor tumor tolerance. Consequently, strategies that target MDSCs hold great potential for enhancing the effectiveness of immune‐based cancer therapies.

In this study, the absence of immune cells lacking CD300ld was particularly noteworthy in tumor environments, implying that cells expressing CD300ld could be facilitators of tumor growth. Notably, CD300‐ld expression was predominantly observed in neutrophils and was markedly increased following tumor implantation. The role of CD300ld in mediating immune suppression seems to be critical for both tumor development and progression. This finding was further substantiated in CD300ld‐knockout mice, which demonstrated that CD300ld acts as a crucial functional receptor on neutrophils/PMN‐MDSCs to promote tumor growth. Comparative studies revealed that CD300ld knockout mice experienced a considerable decrease in tumor size relative to their wild‐type counterparts, underscoring the significance of CD300ld on neutrophils for tumor‐driven immune suppression.

To investigate the role of CD300ld in regulating tumor immunity, researchers initially examined the impact of CD300ld deletion on the tumor microenvironment. The absence of CD300ld led to a marked reduction in the accumulation of PMN‐MDSCs within tumors, coupled with a notable increase in the infiltration of CD8+ T cells. Therefore, CD300ld deletion can reduce the immunosuppressive capacity of the tumor microenvironment. These findings further underscore the pivotal function of PMN‐MDSCs in shaping the immunological landscape of tumors.

CD300ld plays a dual role in the functionality of PMN‐MDSCs: it governs their recruitment to the tumor site and their capacity to robustly inhibit immune effector cells. PMN‐MDSCs deficient in CD300ld‐related genes exhibited diminished migration and recruitment to the tumor locale. Concurrently, their capacity to suppress T‐cell proliferation was significantly reduced. Further investigations revealed that S100A8/A9 is the critical downstream effector molecule affected by CD300ld. The activation of STAT3 by CD300ldCD300ld elevates the expression of S100A8/A9, establishing the CD300ld‐STAT3‐S100A8/A9 axis. This axis is instrumental in orchestrating the recruitment and immunosuppressive function of PMN‐MDSCs.

Subsequent to the establishment of tumors, researchers explored the potential antitumor effects of inhibiting CD300ld. Their findings revealed that conditional knockout of CD300ld markedly hindered tumor progression postestablishment. Additionally, administering the extracellular region of the CD300ld protein as a competitive inhibitor also significantly impeded the progression of both secondary and primary tumors. When combined with Programmed death‐1 (PD‐1) blockade, CD300ld, and PD‐1 dual blockade resulted in more effective combination therapy. Consequently, targeting CD300‐ld represents a promising antitumor therapeutic strategy and has the potential to significantly enhance the efficacy of existing ICB therapies.

To further investigate the role of human CD300ld in cancer, researchers searched The Cancer Genome Atlas database and examined samples from patients with various tumor types. The results showed that inducing CD300ld knockout after tumor establishment could significantly inhibit tumor development. Moreover, the expression level of CD300ld in tumor tissues was significantly greater than that in adjacent nontumor and normal tissues and correlated strongly with neutrophil infiltration and patient prognosis. The team went on to create CD300ld humanized mice, which provided further evidence that human CD300ld shares functional homology with its mouse counterpart. Competitive inhibition of human CD300LD also demonstrated significant antitumor effects. The tumor‐promoting role of CD300ld appears to be conserved between mice and humans, indicating that CD300ld could be a viable target for cancer immunotherapy in humans.

Identifying molecular targets of PMN‐MDSCs and understanding the underlying mechanisms have recently attracted increasing interest. In addition to CD300ld, several other molecules have been reported to participate in PMN‐MDSC regulation. Moreover, PMN‐MDSCs have potential as therapeutic targets for cancer immunotherapy.

In a study of molecules that regulate migration, Hawila et al. reported that CCR5 can direct the mobilization of polymorphonuclear myeloid cells from the bone marrow to the blood to support tumor development.^3^ In immunosuppression studies, Veglia and colleagues reported that mouse and human PMN‐MDSCs upregulate fatty acid transport protein 2 (FATP2). Deletion of FATP2 abrogated the suppressive effect of PMN‐MDSCs. Inhibition of FATP2 abrogated the activity of PMN‐MDSCs and delayed tumor progression. In combination with checkpoint inhibitors, FATP2 inhibition blocked tumor progression in mice. FATP2 represents a target for selectively inhibiting the functions of PMN‐MDSCs and improving the efficacy of cancer therapy.^4^ Chen et al. discovered that the glycoprotein olfactomedin‐4 (OLFM4) was highly expressed in PMN‐MDSCs in colorectal cancer patients. Moreover, mice lacking OLFM4 in myeloid cells exhibited poor recruitment of PMN‐MDSCs and an increased response to anti‐PD1 therapy. The main mechanism of OLFM4‐mediated PMN‐MDSC activity involves the OLFM4/NF‐kappaB/PTGS2 pathway, which promotes PMN‐MDSC recruitment.^5^

Overall, this study highlights the potential of CD300ld as a target for cancer immunotherapy. Through the work of Wang and colleagues, there is now a better understanding of the role of CD300ld as a key immune suppressor on PMN‐MDSCs, contributing to tumor immune evasion and offering a molecular basis for its targeting in cancer immunotherapy strategies.^1^, ^2^ While the evidence presented reinforces the necessity of CD300ld in the immunosuppressive activities of PMN‐MDSCs, additional studies have identified other molecules in PMN‐MDSCs that are also instrumental in mediating their recruitment and immunosuppressive functions. These findings suggest the presence of a complex network of factors that may need to be considered when developing comprehensive immunotherapeutic approaches (Figure 1).

**FIGURE 1:**
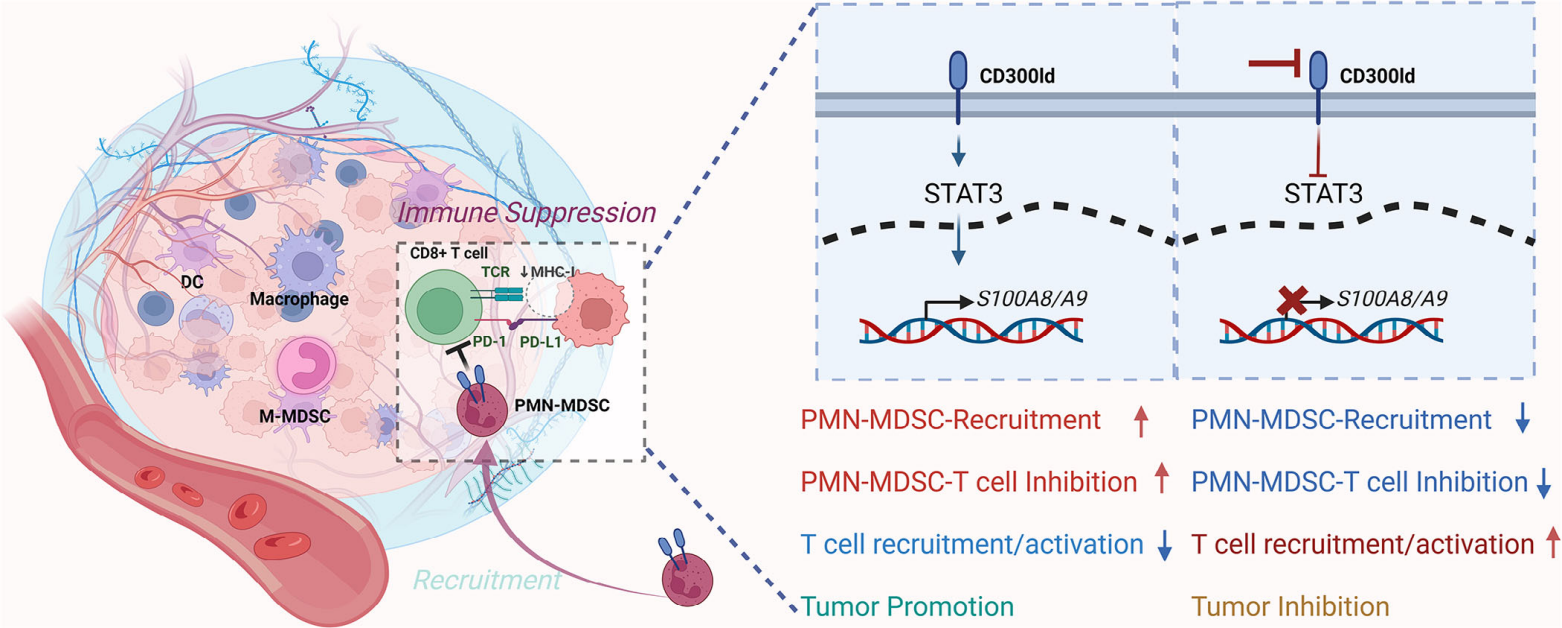
CD300ld plays an integral role in PMN‐MDSCs promoting tumor‐mediated immunosuppression.S100A8/A9 is a key downstream effector molecule mediated by CD300ld. Activation of STAT3 by CD300ld can improve the expression of S100A8/A9 and establish the CD300Ld‐STAT3‐S100A8 /A9 axis.

## AUTHOR CONTRIBUTIONS

Shiqi Li drafted the manuscript and constructed the figure. Xinghua Long drafted and reviewed the manuscript. All the authors read and approved the final manuscript.

## CONFLICT OF INTEREST STATEMENT

The authors declare no conflict of interest.

## ETHICS STATEMENT

Not applicable.

## Data Availability

Not applicable.
